# Beyond BMI: A Cross-Sectional Analytical Study of the Association Between Body Composition Parameters and Functional Disability in Overweight and Obese Adults With Chronic Low Back Pain

**DOI:** 10.7759/cureus.103765

**Published:** 2026-02-17

**Authors:** Venkat N Teja, Rajasegaran Rajalakshmi, Praveen Prakash, Ramesh AS, Deep Sharma

**Affiliations:** 1 Department of Physiology, Jawaharlal Institute of Postgraduate Medical Education and Research, Puducherry, IND; 2 Department of Neurosurgery, Jawaharlal Institute of Postgraduate Medical Education and Research, Puducherry, IND; 3 Department of Orthopedics, Jawaharlal Institute of Postgraduate Medical Education and Research, Puducherry, IND

**Keywords:** body composition, body mass index (bmi), chronic low back pain (clbp), lean body mass, obesity

## Abstract

Background

Low back pain (LBP) is a significant cause of global disability and is closely associated with overweight and obesity due to increased mechanical load, chronic inflammation, and altered muscle function. The body mass index (BMI) fails to differentiate between fat and lean compartments, thereby limiting its clinical utility. Evidence on the association between specific body composition parameters, such as body fat mass index (BFMI), fat-free mass index (FFMI), and lean body mass (LBM), and functional disability in the Indian population remains scarce.

Materials and methods

This cross-sectional study included 99 overweight and obese adults with chronic LBP attending a tertiary care teaching hospital in South India, recruited through convenience sampling. Body composition was assessed using bioelectrical impedance analysis, and functional disability was evaluated with the Oswestry Disability Index (ODI). Pearson correlation and linear regression analyses were performed to assess associations between body composition parameters and disability.

Results

The mean age of participants was 47.6 ± 9.5 years, with females comprising 94.9% of the sample. Obesity was prevalent in 70.7% of participants, and most reported severe pain with long symptom duration. Severe functional disability was observed in 68.7% of cases. ODI showed strong negative correlations with LBM (r = -0.67*,* p < 0.001) and FFMI (r = -0.67, p < 0.001), and a strong positive correlation with BFMI (r = 0.74, p < 0.001). A multiple linear regression model including BFMI and FFMI explained 69% of the variance in ODI scores.

Conclusions

Body composition parameters are strongly associated with functional disability in overweight and obese adults with LBP. Higher adiposity is linked to greater disability, whereas higher lean mass is protective. Assessing body composition beyond BMI may aid clinical decision-making and inform targeted lifestyle and rehabilitative interventions.

## Introduction

Low back pain (LBP) remains a highly prevalent musculoskeletal condition globally and is consistently ranked as the leading cause of years lived with disability across all regions and age groups [1]. Its burden spans the adult lifespan and places substantial strain on health systems, particularly at the primary care level, where the majority of individuals with LBP are initially managed. Degenerative changes of the lumbar spine, including intervertebral disc degeneration, are frequently implicated in chronic LBP and contribute to functional limitations that adversely affect quality of life and work productivity [2].

The state of being overweight or obese has been increasingly recognized as an important modifiable risk factor for the development, persistence, and severity of LBP. Numerous epidemiological and clinical studies have demonstrated strong associations between excess body weight and lumbar disc degeneration, as well as with the occurrence and chronicity of LBP [3]. The mechanisms underlying this association are thought to be multifactorial. Biomechanically, increased body mass and, in particular, central adiposity result in greater axial loading, thereby altering spinal biomechanics. This can accelerate the degenerative processes occurring within intervertebral disks [3]. It has also been established that adipose tissue is metabolically active, releasing pro-inflammatory adipokines and cytokines, which can accelerate degeneration, heighten pain sensitivity, and impair tissue repair [4].

According to Indian consensus guidelines, overweight is defined as a body mass index (BMI) of 23.0-24.9 kg/m², and obesity is defined as a BMI of 25 kg/m² or greater [5]. While BMI is widely used due to its simplicity, it provides only a crude measure of body size and does not differentiate between fat mass and lean tissue compartments. This limitation is particularly relevant in musculoskeletal disorders such as LBP, where adiposity and muscle mass may exert distinct and even opposing effects on pain, spinal stability, and functional capacity [6,7]. Consequently, relying solely on BMI may obscure clinically meaningful associations between body composition and disability.

Body composition assessment evaluates body weight by distinguishing its core components, including fat mass, lean tissue, minerals, and body water. Unlike BMI, body composition assessment offers a clinically meaningful perspective by distinguishing between adiposity and lean tissue [8].

Bioelectrical impedance analysis (BIA) is a widely used, noninvasive, and cost-effective method for assessing body composition and has been applied across clinical and epidemiological settings. Although it is not as accurate as reference methods such as dual-energy X-ray absorptiometry (DEXA) and magnetic resonance imaging (MRI), it has been shown to have sufficient cross-sectional accuracy for clinical practice reliability [9,10]. Its substantially lower cost makes it more feasible to obtain, particularly outside tertiary care settings [10].

Functional disability related to LBP is commonly assessed using the Oswestry Disability Index (ODI), a validated, disease-specific self-report instrument that quantifies disability across activities of daily living [11]. The ODI is extensively validated and remains one of the most widely accepted outcome measures for quantifying disability in individuals with chronic LBP, making it particularly suitable for both clinical practice and research settings.

Despite the high prevalence of both obesity and chronic LBP in India [12], there is a paucity of evidence examining the association between detailed body composition parameters and functional disability in clinical populations. Most existing studies continue to rely on BMI as the primary indicator of obesity, thereby failing to capture the differential contributions of adiposity and lean mass to pain-related disability. This gap is especially relevant in tertiary care settings, where individuals often present with persistent symptoms and significant functional impairment.

Therefore, the primary objective of this study was to determine the association of body composition parameters (body fat mass index (BFMI), fat-free mass index (FFMI), and lean body mass (LBM)) with functional disability, as measured by the ODI, among overweight and obese adults with chronic LBP attending a tertiary care hospital in South India.

## Materials and methods

This cross-sectional observational study was conducted between May and August 2025 among ambulatory adults with chronic low back pain who attended the outpatient services of the Departments of Neurosurgery and Orthopaedics at the Jawaharlal Institute of Postgraduate Medical Education and Research, Puducherry, India. The study was approved by the Institutional Ethics Committee for Observational Studies (approval number: JIP/IEC-OS/2024/849) and was conducted in accordance with the principles outlined in the Declaration of Helsinki. The study details were explained to the participants, and written informed consent was obtained from all participants prior to enrollment. For participants with limited literacy, study information was provided in their local language, and consent was obtained via a thumbprint in the presence of an impartial witness, in accordance with institutional ethics committee guidelines and the International Committee of Medical Journal Editors (ICMJE) recommendations.

The objective of this study was to investigate the association between BFMI, LBM, FFMI, and functional disability as assessed by the ODI.

Sample size estimation, power, and sampling

The sample size of 99 was initially calculated for a parent study comparing body fat percentage between patients with mild/moderate and severe functional disability, based on assumptions drawn from the existing literature [13]. The present manuscript focuses on a secondary objective, examining the associations between continuous variables. With the final sample size of N = 99, the study had adequate statistical power to detect moderate correlations (correlation coefficient r ≥ 0.36) at a 5% significance level and to perform multiple linear regression analyses with up to four predictor variables, assuming a moderate effect size (Cohen's f² = 0.20), which is sufficient for the present analysis.

Participants were recruited via convenience sampling from the study center's outpatient services. Of the 280 patients screened, 99 met eligibility criteria and consented to participate in the study.

Inclusion and exclusion criteria

Individuals aged 40-60 years, classified as overweight (BMI 23.0-24.9 kg/m²) or obese (BMI ≥25 kg/m²), presenting with chronic LBP of at least three months' duration, with an intensity score ≥4 on the Numeric Rating Scale (NRS; 0-10), and radiological confirmation of degenerative lumbar spine disease (X-ray/CT/MRI) were included. Patients were excluded if they had literacy or language difficulties since the ODI is a self-administered questionnaire that requires independent reading and comprehension to ensure accurate responses. Including patients with literacy or language difficulties would have required interviewer assistance, potentially introducing bias and affecting the tool’s psychometric validity. Therefore, this exclusion was applied to maintain data quality and internal validity. Further, any patient with a history of psychiatric disorders, congenital spinal disease, ligament ossification, diffuse idiopathic skeletal hyperostosis, a definite indication for urgent surgery (e.g., cauda equina syndrome), a history of prior spinal surgery, presence of orthopedic metal implants (except titanium) in any body region, or a cardiac pacemaker/implantable cardioverter-defibrillator.

Assessment of functional disability

Functional disability in activities of daily living was measured using a validated Tamil version of the ODI [14,15]. This self-administered questionnaire comprises 10 sections, each scored on a 0-5 scale, with 5 indicating maximum disability. The ODI percentage was calculated as ODI score = (sum of item scores / total possible score) × 100. Disability categories were defined as minimal (0-20%), moderate (21-40%), severe (41-60%), crippled (61-80%), and complete (81-100%) [14,15]. The ODI demonstrates excellent internal consistency (Cronbach's alpha = 0.92), test-retest reliability (intraclass correlation coefficient = 0.92), and construct validity [15].

Assessment of body composition

Body composition analysis was performed in the Department of Physiology using the QuadScan 4000 BIA device (BodyStat Ltd., Isle of Man) according to the standard protocol. The instrument was calibrated according to the manufacturer's defined calibration protocol prior to measurements. Participants were placed in the supine position, with two current-introducing electrodes attached to the dorsum of the right hand and foot, and two voltage-sensing electrodes placed on the right wrist and ankle. After entering the anthropometric details, the analyzer measured several indices, including body fat percentage, LBM, BFMI, and FFMI. Measurements were performed under standardized conditions, with participants instructed to refrain from consuming heavy meals and engaging in strenuous physical activity for at least two hours prior to assessment.

Statistical analysis

Data entry was performed using EpiCollect, and statistical analysis was conducted using SPSS Statistics version 19 (IBM Corp. Released 2010. IBM SPSS Statistics for Windows, Version 19.0. Armonk, NY: IBM Corp.). Sociodemographic and clinical variables (age, sex, height, weight, BMI, NRS score, pain duration, functional disability, and body composition indices) were analyzed. Continuous variables were assessed for normality using the Shapiro-Wilk test and expressed as mean ± standard deviation or median (interquartile range), as appropriate. Categorical variables were summarized as frequencies and percentages.

Associations between ODI scores and body composition parameters were assessed using Pearson's correlation for normally distributed variables and Spearman's rank correlation for non-normally distributed variables. Variables showing significant correlations were entered into simple and multiple linear regression models to examine their association with functional disability.

Prior to multiple linear regression, assumptions of linearity, normality of residuals, homoscedasticity, and absence of multicollinearity were evaluated. Multicollinearity was assessed using the variance inflation factor, and variables with significant collinearity were excluded from the final model. All statistical tests were two-tailed, and a p-value <0.05 was considered statistically significant.

## Results

A total of 99 overweight and obese adults with chronic LBP were included in the analysis. Sociodemographic (age, sex, occupation) and clinical characteristics (body composition parameters, type of functional disability, ODI score) were collected.

Sociodemographic characteristics

The mean age of the study participants was 47.6 ± 9.5 years. The cohort was predominantly female (94.9%). Most participants were classified as obese (70.7%), while the remainder were overweight. A substantial proportion of participants were unemployed or engaged in unskilled occupations. Metabolic comorbidities, including hypertension (18.2%) and diabetes mellitus (12.1%), were present in a minority of participants. Detailed sociodemographic, anthropometric, and comorbidity characteristics are presented in Table 1.

**Table 1:** Sociodemographic, anthropometric, and comorbidity characteristics of the study participants (N = 99) Continuous variables, namely age, height, and weight, are reported as mean ± SD, with a total of 99 participants. Categorical variables, namely gender, occupation, comorbidities, and BMI-based classification into overweight and obese groups, are represented as frequencies. SD: standard deviation, BMI: body mass index

Characteristics	Mean ± SD or frequency (%) for N = 99
Age	47.63 ± 9.52
Gender	
Female	94 (94.9%)
Male	5 (5.1%)
Occupation	
Unemployed	59 (59.6%)
Unskilled	26 (26.3%)
Skilled	10 (10.1%)
Professional	4 (4%)
Comorbidities	
Diabetes mellitus	12 (12.1%)
Hypertension	18 (18.2%)
Hypothyroidism	2 (2%)
Renal disease	1 (1%)
Heart disease	1 (1%)
Anthropometry	
Height in cm	154.04 ± 7.01
Weight in Kg	65.81 ± 9.70
BMI	
Overweight	29 (29.3%)
Obese	70 (70.7%)

Pain characteristics of the study participants

Most participants (88.9%) reported severe pain, with a mean NRS score of 8 ± 1. Pain duration varied across the cohort, with many participants reporting symptoms for more than one year. Pain characteristics are summarized in Table 2.

**Table 2:** Pain characteristics of the study participants (N = 99) Continuous variables, namely the mean NRS score, are presented as mean ± SD. Categorical variables, namely severity groups based on NRS and duration of pain, are represented as frequencies. NRS: Numeric Rate Scale, SD: standard deviation

Parameter	Mean ± SD or frequency (%) for N = 99
Severity groups based on NRS	
Moderate	11 (11.1 %)
Severe	88 (88.9 %)
Duration of pain	
Less than 1 year	22 (22.2 %)
1-3 years	33 (33.3 %)
3-5 years	23 (23.2 %)
5-7 years	12 (12.1 %)
7-10 years	1 (1 %)
Above 10 years	8 (8.1 %)
Mean NRS score	
NRS score (mean ± SD)	8 ± 1

Functional disability among the study participants

Functional disability, assessed using the ODI, was predominantly severe. Overall, 68.7% of participants demonstrated severe disability, while 31.3% had mild to moderate disability. The mean ODI score for the cohort was 58 ± 17. The distribution of functional disability is shown in Table 3.

**Table 3:** Functional disability as measured by the ODI in the study participants (N = 99) Continuous variable, namely the mean ODI score, is reported as mean ± SD. The categorical variable, namely the severity classification based on the ODI score, are represented as frequencies. ODI: Oswestry Disability Index

Parameter	Value
Classification based on ODI score	
Mild/moderate (frequency (%))	31 (31.3%)
Severe (frequency (%))	68 (68.7%)
ODI score as a continuous variable	
Mean ODI score (mean ± SD)	58 ± 17

Correlation analysis between the ODI and body composition parameters

Correlation analysis demonstrated significant associations between functional disability and body composition parameters (Table 4). A significant inverse correlation was found between LBM and ODI scores (r = -0.67, p < 0.001). Similarly, a significant inverse correlation was also observed between FFMI and ODI scores (r = -0.67, p < 0.001). In contrast, BFMI demonstrated a strong positive correlation with ODI scores (r = 0.74, p < 0.001). No significant correlation was observed between BMI and ODI scores.

**Table 4:** Correlation between patient ODI and body composition (N = 99) * Statistically significant at p-value < 0.001 BMI: body mass index, BFMI: body fat mass index, FFMI: fat-free mass index, ODI: Oswestry Disability Index, LBM: lean body mass

Variable correlated to ODI score	Mean ± SD (N = 99)	Pearson correlation coefficient (ρ)	p-value
BMI	27.81 ± 3.97	0.11	0.282
LBM	60.6 ± 2.74	-0.67	<0.001*
BFMI	10.0 ± 1.87	0.74	<0.001*
FFMI	12.9 ± 0.53	-0.67	<0.001*

Linear regression analysis of ODI with body composition parameters

Simple linear regression analyses, with the ODI score as the dependent variable and LBM, BFMI, and FFMI as independent variables, showed statistically significant associations for each model individually (Table 5). Collinearity analysis revealed significant collinearity between LBM and FFMI; therefore, LBM was excluded from the multiple linear regression model.

In the final multiple linear regression model, BFMI and FFMI together accounted for 69% of the variance in ODI scores (R² = 0.69, p < 0.001).

**Table 5:** Linear regression analysis between ODI and body composition parameters * Statistically significant at p < 0.001 ODI: Oswestry Disability Index, BMI: body mass index, BFMI: body fat mass index, FFMI: fat-free mass index

Response variable	Predictor variables	Fitted regression equation	R^2^	F	β	95% CI (lower limit, upper limit)	p-value
ODI score	Lean mass	ODI = 311.86 – (4.260 × lean mass)	0.47	F (1, 97) = 87.32	–0.69	Lean mass: (–5.2, – 3.4), constant: (259.9, 366.9)	<0.001*
ODI score	BFMI	ODI = –14.56 + (6.78 × BFMI)	0.48	F (1, 97) = 124.47	0.75	BFMI: (5.6, 7.9), constant: (–26.8, –2.3)	<0.001*
ODI score	FFMI	ODI = 334.34 – (21.69 × FFMI)	0.47	F (1, 97) = 87.306	–0.69	FFMI: (–26.3, –17.1), constant: (274.6, 394.1)	<0.001*
ODI score	BFMI, FFMI	ODI = 173.08 + (4.87× BFMI) – (13.01 × FFMI)	0.69	F (2, 96) = 105.75	–0.41 (FFMI), 0.54 (BFMI)	BFMI: (3.7, 6.1), FFMI: (–17.2, –8.9), constant: (112.3, 233.9)	<0.001*

## Discussion

In this cross-sectional observational study conducted at a tertiary care center in South India, we examined the association between body composition parameters and functional disability among overweight and obese adults with chronic LBP. Using BIA, indices of adiposity and muscle mass, including BFMI, FFMI, and LBM, were assessed and related to disability severity as measured by the ODI. The study included ambulatory patients aged 40-60 years with radiologically confirmed degenerative lumbar spine disease and clinically significant pain, and most participants (94.9%) were female. The mean NRS score across all participants was 8 ± 1, and only 22.2% had a symptom duration of less than one year, suggesting that most had long-standing disease.

This study adds to the existing evidence on the association between body composition and functional disability in overweight and obese patients with LBP. We found that higher BFMI was positively associated with ODI scores, whereas LBM and FFMI were inversely associated with disability. We also found a statistically significant (p < 0.001) multiple linear regression model for ODI score, with FFMI and BFMI as predictors, that explained up to 69% (R² = 0.69) of the variation in ODI score, indicating a strong statistical association within this clinical sample. In this adjusted regression model, higher BFMI was independently associated with increased disability, with each 1 kg/m² increase in BFMI corresponding to a 4.87-point increase in ODI score. Conversely, higher FFMI was associated with lower disability, with each 1 kg/m² increase corresponding to a 13.01-point reduction in ODI.

Although causal conclusions cannot be made, taken together, these results suggest that excess adiposity may exert a detrimental influence on function. At the same time, higher lean mass may be protective against disability. Our findings extend beyond prior work that has relied primarily on BMI. Although epidemiological studies, such as those by Heuch et al., consistently show that higher BMI increases the risk of chronic LBP and symptom persistence [16], BMI represents a crude surrogate measure that does not distinguish between fat and lean compartments.

Using BIA, our study identifies BFMI as a measure that is strongly and significantly correlated with disability, whereas FFMI and LBM are negatively correlated with disability. These findings suggest that individuals with higher lean mass and lower fat mass tend to report lower levels of functional disability, as measured by the ODI. This is in agreement with Hussain et al. [17], who demonstrated that both fat mass and fat distribution are associated with greater LBP intensity and disability, underscoring the limitations of BMI as a clinical marker.

The negative association between lean mass and disability observed in our cohort aligns with emerging mechanistic evidence. Johnson et al. reported that body composition, notably higher lean mass, was independently associated with lower pain sensitivity, regardless of BMI [18]. This supports the possibility that muscle may contribute to spinal stability and load distribution, as well as to resilience against maladaptive pain processing. Beyond mechanical alterations, excess adiposity may contribute to functional disability through biochemical processes, both local and systemic. Adipose tissue functions as an endocrine organ that releases pro-inflammatory cytokines like TNF-α and IL-6, as well as adipokines like leptin [19]. Adipokines have been shown to modulate immune responses, promote catabolic changes in intervertebral disc tissues, and trigger pro-inflammatory cascades that, in turn, can sensitize nociceptors and impair tissue repair mechanisms [19]. This forms a link between adiposity and disability that is independent of mechanical load. Clinically, this implies that interventions aimed at preserving or augmenting lean mass, while also reducing adiposity, may improve function and reduce disability in obese patients with LBP.

Interestingly, the correlation magnitudes in our study (e.g., BFMI r = 0.74; FFMI r = -0.67) are larger than those reported in recent systematic reviews and meta-analyses. A meta-analysis by Liechti et al. found only modest associations between body composition and pain intensity in adults with chronic nonspecific LBP [20]. Several contextual factors may contribute to this discrepancy. The study group comprised predominantly obese, middle-aged women (94.9%), a group commonly encountered in clinical practice. Women generally have higher body fat percentages and lower absolute lean mass than men at comparable BMI levels, which may predispose them to greater functional impairment for a given degree of excess weight [21].

Furthermore, the age range of our cohort (40-60 years) overlaps with the perimenopausal period, during which estrogen decline is associated with unfavorable changes in body composition, including increased fat mass and accelerated loss of skeletal muscle [22,23]. These changes have been linked to reduced muscle strength, impaired postural control, and greater susceptibility to musculoskeletal pain and disability [23]. Moreover, our primary outcome, the ODI, reflects functional limitation rather than pain intensity, which may yield stronger associations. Selection bias in a clinic-based cohort, where individuals with greater disability are more likely to seek care, may also have amplified the observed effect sizes and should be considered when interpreting the magnitude of the observed associations. While these differences warrant cautious interpretation, they lend support to the biological plausibility of body composition as a meaningful determinant of disability.

From a translational perspective, these findings highlight the potential relevance of integrated interventions that target both adiposity reduction and lean mass preservation. Evidence suggests that caloric restriction alone can lead to loss of both fat and lean compartments, whereas combining nutritional counselling with resistance training optimizes fat reduction while maintaining or increasing muscle mass [24]. Such body composition-focused strategies may offer advantages over approaches that focus primarily on BMI reduction. Furthermore, incorporating body composition metrics into routine evaluation provides more actionable insights for tailoring rehabilitation and weight-management programs than BMI alone.

Limitations and future directions

This study has the following limitations: first, causal inferences cannot be made due to the cross-sectional study design. Although our findings are consistent with prior evidence that fat mass and distribution contribute to disability [12], prospective studies are needed to confirm the directionality of this relationship. Second, BIA, while practical and widely used, is less precise than imaging modalities such as DEXA or MRI, particularly in obese individuals. Advanced imaging methods could clarify the roles of visceral, subcutaneous, and intermuscular fat compartments, as highlighted in the meta-analysis by Liechti et al. [20]. Third, the cohort was overwhelmingly female, which may limit the generalizability of the findings to male populations. It was also prone to selection bias due to convenience sampling in a tertiary care setting, which warrants caution when generalizing. Fourth, we did not assess muscle function (e.g., strength, endurance), which may provide complementary insight, as lean tissue has been linked to reduced pain sensitivity [18]. Finally, residual confounding from unmeasured factors such as physical activity, psychological stress, or socioeconomic status cannot be ruled out. The relatively high proportion of cases with severe disability could also have contributed to high effect sizes. Future research may prioritize longitudinal cohort studies assessing whether changes in BFMI and FFMI predict disability trajectories over time. Randomized controlled trials should evaluate combined nutritional and resistance-training interventions, with outcomes including body composition, muscle function, inflammatory biomarkers, and validated disability measures. Sex-balanced cohorts and mediation analyses that distinguish between biomechanical and inflammatory pathways would refine mechanistic understanding and support precision rehabilitation strategies.

Clinical implications

Despite these limitations, this study highlights the potential role of body composition in shaping functional disability among obese patients with LBP. Consistent with Hussain et al. [17], excess adiposity appears to exacerbate disability, whereas lean mass is protective, as also supported by Johnson et al. [18]. Although pooled estimates from the meta-analysis by Liechti et al. [20] suggest only modest overall effects, our findings highlight that in high-risk subgroups such as obese middle-aged women, body composition exerts a clinically significant impact. Clinicians may therefore consider moving beyond BMI and incorporating body composition assessments into the standard evaluation of patients with LBP, thereby tailoring management accordingly. Interventions that integrate resistance training with nutritional optimization may help address adiposity and preserve lean mass.

## Conclusions

In overweight and obese patients with LBP, functional disability shows a close association with body composition parameters, with higher adiposity and lower lean mass being linked to greater levels of disability. These findings support the inclusion of body composition assessment as an adjunct to BMI in the clinical evaluation of patients with LBP. A multidisciplinary approach integrating nutrition and exercise strategies may help optimize functional outcomes in this high-risk population. Tailoring management plans to individual body composition profiles may enable more precise and impactful rehabilitation in this high-risk group.
